# Etiology and pattern of maxillofacial fractures among patients who visited Jimma Medical Center Dental Clinic, Jimma, Southwest Ethiopia

**DOI:** 10.1097/MD.0000000000042569

**Published:** 2025-06-06

**Authors:** Gemechu Tola Wayiso, Fikadu Seyoum Tola, Midekso Sento Erba, Diliab Desta

**Affiliations:** a Department of Clinical Anatomy, College of Medicine and Health Sciences, Ambo University, Addis Ababa, Ethiopia; b Department of Medical Biochemistry, College of Medicine and Health Sciences, Ambo University, Addis Ababa, Ethiopia; c Department of Clinical Anatomy, Adama Hospital Medical College, Addis Ababa, Ethiopia; d Department of Biomedical Sciences, Faculty of Medical Science, Jimma University, Jimma, Ethiopia.

**Keywords:** clinical outcome, Ethiopia, head injury, Jimma, maxillofacial fracture, road traffic accident, surgical site infection

## Abstract

The maxillofacial region consists of soft and hard tissues that form the face and extend from the frontal bone superiorly to the mandible inferiorly. Because the face is the most exposed part of the body, it is especially vulnerable to trauma. Trauma to the maxillofacial regions is a major public health problem worldwide. Nearly 5% to 10% of trauma patients have facial fractures. Thus, the aim of this study was to assess the etiology and pattern of maxillofacial fractures among patients who visited Jimma Medical Center (JMC) dental clinic, Southwest Ethiopia. Institutional based retrospective cross-sectional study was conducted on 331 patients (279 males and 52 females with a mean age of 26.23 ± 13.51 years) with maxillofacial fractures who visited JMC dental clinic from January 2018 to December 2020. To collect data first charts of the patients were found using their medical record numbers. Then information like socio-demographic characteristics, patterns of fractures, and causes of fractures, was collected using a structured and pretested checklist from the chart. Data was entered into the Epi-data version 3.1 and exported to SPSS version 25 for analysis. Descriptive analysis was done and presented by the use of tables, bar graphs and pie chart. The leading cause of fracture was road traffic accidents (45%), followed by assault (33.2%) and accidental fall (11.8%). Head (51.6%) injuries were the most common associated injuries. Surgical site infection (52.2%) was the most common posttreatment complication. The study’s findings can be used to guide public health activities, healthcare professional training, and resource allocation in Ethiopia in order to enhance maxillofacial fracture prevention, management, and outcomes.

## 1. Introduction

The maxillofacial region consists of soft and hard tissues that form the face and extend from the frontal bone superiorly to the mandible inferiorly.^[1]^ It consists of the frontal, nasal, palatine, zygomatic, maxillary, and mandibular bones. It divided into 3 sections for detecting trauma to this area: the upper third, the middle third, and the lower third. The lower third of the face is formed by the mandible along with its dentoalveolar arch. Depending on the location of the fracture lines, mandibular fractures are classified as symphyseal, parasymphyseal, body, angle, ramus, coronoid process, and condylar process fractures. The middle third of the face is defined as the area between the supraorbital margins and the occlusal plane of the upper teeth. It is made up of nasal, palatine, zygomatic, and maxillary bones. According to the Le Fort classification, maxillary fractures are classified as Le Fort I, Le Fort II, Le Fort III, and maxillary dentoalveolar fractures. Based on the location of the fracture lines, zygomatic fracture is classified as zygomaticomaxillary complex fracture or zygomatic arch fracture (when only the arch is involved). The upper third of the face is the area above the supraorbital margins. It is formed by the frontal bone.^[2,3]^

The Arbeitsgmeinschaft fur Osteosynthesis (AO) or German “Association for the study of internal fixation” group proposed a system for classifying craniomaxillofacial fractures in adults with 3 levels of precision to describe these injuries in terms of complexity and details. Level 1 is the most basic; it only indicates whether or not fractures exist in 4 anatomical units: the mandible, midface, skull base, and cranial vault. Level 2 describes the fractures’ precise location within the mandible, central and lateral midface, internal orbit, endocranial and exocranial skull base, and cranial vault. Level 3 provides even more information about the injury’s location, focusing on morphology (fragmentation, displacement, and bone defects) within specific subregions.^[4]^

The pattern of presentation of facial skeleton fractures varies depending on the etiology of injury. Traffic accidents (including motorcycle, automobile, bicycle, and pedestrian hits), assault, fall from a great height, stumbling, sports, industrial/work-related accidents, and others/miscellaneous are common causes of facial bone fractures.^[5]^

The diagnosis of maxillofacial fracture necessitates both clinical and imaging examinations. A history of injury to the area, pain, abnormal mobility, bleeding, crepitus, deformity, ecchymosis, edema, loss of function or interference with function are clinical features of facial fracture.^[6]^ Radiological examination requires lateral skull view, posteroanterior view, and Towne view, orthopantomogram, or computed tomography scan.^[6]^

Trauma is an inevitable part of human life. It is the world’s fifth leading cause of death and disability, accounting for approximately 5 million deaths each year.^[7]^ Because the face is the most exposed part of the body, it is especially vulnerable to trauma.^[8]^ Trauma to the maxillofacial regions is a major public health problem worldwide. Nearly 5% to 10% of trauma patients have facial fractures. They impose a public health burden in terms of workload, time consumed, treatment costs, and psychological effects on victims.^[9]^ It has been claimed that maxillofacial fractures vary greatly between countries and even within the same country. This significant variation can be attributed to a variety of factors, including the population’s age and gender distribution, as well as socioeconomic, cultural, and environmental factors.^[10]^

The majority of maxillofacial fractures occur in people between the ages of 21 and 30 and more common in men, with a male-to-female ratio ranging from 2:1 to 11:1.^[11–13]^ Several studies conducted in various countries of the world shows male predominance with high male-to female ratio. The male-to female ratio in study conducted in Kuwait^[14]^ and Italy^[15]^ was 5.4:1, each respectively, and it is 6.6:1 in study conducted in Punjab, India.^[16]^ However, the ratio was low (2.1:1 and 1.6:1, respectively) according to study conducted in Austria^[13]^ and Canada.^[17]^

According to an Ethiopian study on maxillofacial trauma, only 49.4% of patients suffered soft tissue injury, while the remaining majority (50.61%) sustained maxillofacial bone fractures. Despite the increasing frequency of morbidity and mortality associated with maxillofacial fractures, little is known about maxillofacial fracture in Ethiopia. This issue was previously addressed in a study^[18]^ conducted in the northwestern part of Ethiopia on maxillofacial trauma as a general (not specifically on maxillofacial fractures).

However, because trends in maxillofacial trauma are heavily influenced by time and location, and Ethiopia is a vast country with diverse ethnic, cultural and environmental backgrounds, comprehensive data in the remaining parts of the country is lacking. There has been no previous published research on maxillofacial bone fractures in Jimma where this study is carried out. This study aims to provide recent information on the etiology and pattern of maxillofacial fractures among patients presented at Jimma Medical Center (JMC), Southwest Ethiopia.

## 2. Hypothesis

H0: there is no statistically significant difference in the prevalence of fracture between the 2 sex groups or age groups.

HA: there is a statistically significant difference in the prevalence of fracture between the 2 sex groups or age groups.

## 3. Material and methods

### 3.1. Study design, area and period

Institutional based retrospective cross-sectional study was conducted on patients with maxillofacial bone fracture who visited JMC dental clinic from January 2018 to December 2020. Data was collected from October 1 to 20, 2021. JMC is found in Jimma town which is located in the southwestern part of Ethiopia, 356 km away from Addis Ababa, Ethiopia. It is one of the oldest hospitals in Ethiopia and it is one of the teaching and referral hospitals in Southwest Ethiopia with 800 bed capacities and a catchment population of over 15 million people. The dental clinic of JMC provides services under different departments such as oral and maxillofacial department, general dentistry, endodontic and orthodontics. According to the hospital’s medical record liaison office, about 3632 patients visit the JMC dental clinic each year.

### 3.2. Population

#### 3.2.1. Source population

The source population for this study was all patients who visited JMC dental clinic between January 2018 and December 2020.

### 3.3. Sample size determination

There was no sample size calculation because the entire study population was included as a study subject. All patients who were treated for maxillofacial bone fractures at the JMC dental clinic between January 2018 and December 2020 and whose charts met the inclusion criteria were taken as valid study subjects.

### 3.4. Data collection tool and procedure

Data was collected using a structured questioner which was adapted by extensive review of related studies^[7,9,12,19–21]^ and modified accordingly to meet the study’s objectives. medical record numbers were first obtained from the health management information system registration book in order to obtain the patient’s main file from the chart room. Following that, in the patient’s chart room, the necessary information was searched for in terms of age, gender, anatomical site of fracture, nature of fracture, affected side, number of fractured bone, causes of fractures, associated injuries, and posttreatment complications from the identified charts. Finally, cards containing all variables for the study were identified and data was extracted from them. Two Bachelor of Science (BSc) nurses were assigned to collect data from medical charts, with 1 BSc nurse supervising data collectors throughout the process. The principal investigator (PI) provided timely supervision throughout the data collection period.

### 3.5. Data quality control

Initially, a pretest was performed on 5% of the total patients’ cards prior to the actual data collection time to assess the integrity of an adapted data collection tool. After the pretest, some modifications were made to the tool. To ensure the quality of the data, a 1-day orientation on how to use the data extraction tool was given to 2 data collectors and 1 supervisor prior to data collection. Data collectors checked data for completeness and correctness on a daily basis during collection periods. It was also rechecked daily after collection by the supervisor and PI. During data entry, the data was rechecked again.

### 3.6. Data processing and analysis

The collected data was first checked for completeness. It was then entered into Epi-data version 3.1 and exported to Statistical Package for Social Sciences version 25.0 for analysis. For categorical data, descriptive statistics like frequency and percentage was computed and presented by the use of tables, bar graphs and pie chart. Continuous variables were summarized using means, medians, modes, and standard deviations. The Chi-square (χ^2^) test was used to determine whether there was any relationship between the different categorical variables. At *P*-values of < .05, a statistically significant relationship was declared.

## 4. Results

### 4.1. Socio-demographic characteristics

Only 331 of the 363 medical charts of patients treated for maxillofacial fractures at the JMC dental clinic during the study period were met the inclusion criteria and were analyzed in this study. The study participants comprised of 279 (84.3%) males and 52 (15.7%) females resulting in a male-to female ratio of 5.36: 1.

The patient’s ages ranged from 1 to 70 years with the mean and standard deviations of 26.23 and ± 13.51 years, respectively. The median and the mode of participant’s age were 25 years, each respectively. More than 1-third of the participants 113 (34.1%) were between the ages of 21 and 30. Males sustained maxillofacial bone fractures at a higher rate than females across all age groups, with those aged 21 to 30 being the most affected. Females were more affected in the age range of 0 to 10 (Table 1).

**Table 1:** Age group and sex distributions of maxillofacial fracture patients who visited Jimma Medical Center dental clinic, 2018 to 2020.

Age group	Sex		Frequency (n = 331)	Percentage (%)
	Male	Female		
0 to 10	35	16	51	15.4
11 to 20	64	7	71	21.5
21 to 30	101	12	113	34.1
31 to 40	47	5	52	15.7
41 to 50	17	7	24	7.3
>50	15	5	20	6
Total	279	52	331	100

### 4.2. *Patterns of* fracture

**Figure 1.**
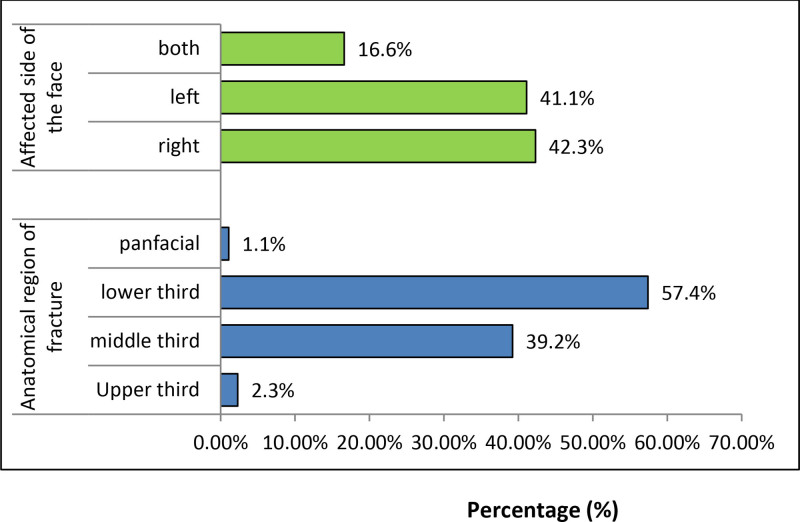
Anatomical region of fractures and affected side of face in maxillofacial fracture patients who visited JMC dental clinic, 2018 to 2020.

Figure 1 shows anatomical region of fractures and affected side of the face. The lower third (mandible) of the face was the most commonly fractured site in this study, sustained by 202 patients, followed by the middle third (138 cases). Only 8 cases had upper third (frontal bone) fractures (Fig. 1).

A total of 324 (64.54%) mandibular fractures were sustained by 202 patients. The most frequently fractured mandibular site was the parasymphysis, accounting for 17.52% of all fractures. It was followed by the body, angle, dentoalveolar process, condylar process, symphysis, ramus and coronoid process fractures, accounting for 13.54%, 9.60%, 7.76%, 7.53%, 6.80%, 1%, and 0.79%) of all fractures. In 138 patients, 166 middle third fractures were observed. The most commonly fractured bone of middle third was zygomatic bone (n = 61, 12.15%), followed by nasal bone (n = 56, 11.15%) and maxilla (n = 34, 6.80%). Fractures of orbital wall, Naso-orbito-ethmoidal and palatine bone were rare in this study, observed only in 4 (0.79%), 6 (1.18%), and 5 (1%), respectively. Regarding the fracture of zygoma, zygomatic complex fractures were the most common, accounting for 8.60% of all fractures, while zygomatic arch fractures were less common, and accounting for only 3.55% of all fractures (Table 2).

**Table 2:** Pattern of maxillofacial fractures in patients who visited Jimma Medical Center dental clinic, 2018 to 2020.

Region	Anatomic site	Frequency	% of all fractures
Upper third	Frontal	8	1.60
Middle third	Nasal	56	11.15
	Palatine	5	1.00
	Naso-orbito-ethmoidal	6	1.18
	Orbital wall	4	0.79
	Orbital blow in	0	0.00
	Orbital blow out	4	0.79
	Zygomatic	61	12.15
	Zygomaticomaxillary complex	43	8.60
	Zygomatic arch	18	3.55
	Maxilla	34	6.80
	Le Fort I	9	1.80
	Le Fort II	14	2.80
	Le Fort III	4	0.79
	Maxillary dentoalveolar	7	1.41
Lower third	Mandibular	324	64.54
	Symphysis	34	6.80
	Parasymphysis	88	17.52
	Body	68	13.54
	Angle	48	9.60
	Ramus	5	1.00
	Coronoid process	4	0.79
	Condylar process	38	7.53
	Dentoalveolar process	39	7.76
Panfacial		4	0.79
Total fracture		502	100

**Figure 2.**
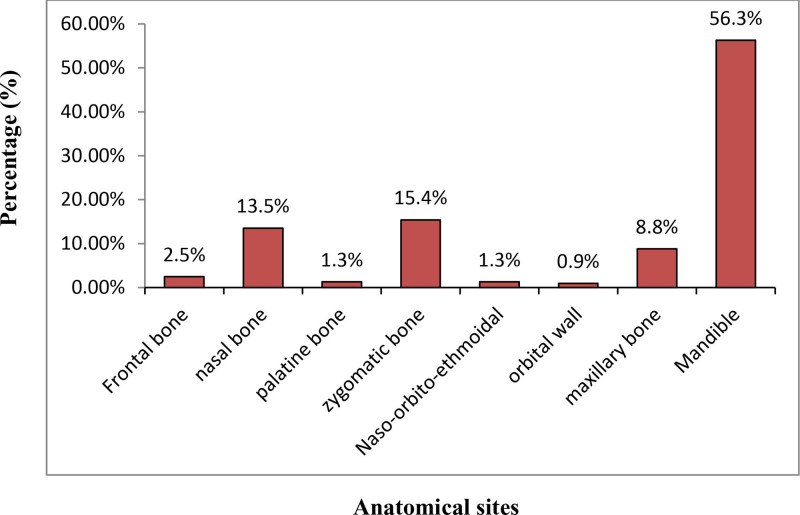
Distribution of maxillofacial bone fractures in male victims who visited JMC dental clinic, 2018 to 2020.

**Figure 3.**
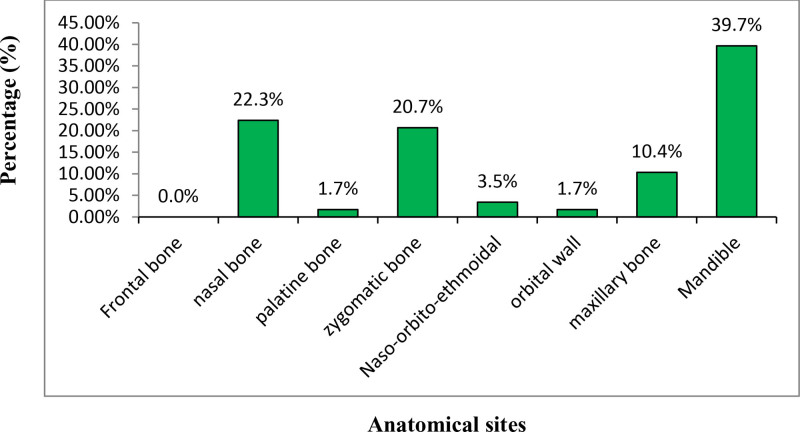
Distribution of maxillofacial bone fractures in female victims who visited JMC dental clinic, 2018 to 2020.

Figure 2 and 3 show the distributions of maxillofacial bone fractures in male and female patients respectively. In male patients, the most commonly fractured bone of facial skeleton was the mandible 179 (56.3%) followed by zygomatic bone 49 (15.4%) while the least fractured bones were naso-orbito-ethmoidal and palatine bone 4 (1.3%) each respectively and orbital wall 3 (0.9%) (Fig. 2).

In female patients, it was observed that the mandible 23 (39.7%) followed by the nasal bone 13 (22.3%) were the most frequently affected bones of facial skeleton while the least affected bones were the orbital wall and palatine bone, each accounted for 1 (1.7%). Frontal bone fracture was not sustained by female victims in this study (Fig. 3).

Table 3 presents the distributions of anatomical sites of fractures across the age groups. In all age groups, the mandible was the most frequently fractured bone. It was followed by nasal bone fractures in the 0 to 10, 11 to 20, and 31 to 40 age groups, zygomatic fractures in the 21 to 30 and 41 to 50 age groups, and maxillary fractures in the over-50 age group. The frontal bone, palatine bone, naso-orbito-ethmoidal bone, and orbital wall, on the other hand, were the least fractured bones across all age groups (Table 3).

**Table 3:** Anatomical site and age distribution of maxillofacial bone fractures in patients who visited Jimma Medical Center dental clinic, 2018 to 2020.

Anatomical site of fracture	Age groups						Total
	0 to 10	11 to 20	21 to 30	31 to 40	41 to 50	>50	
Frontal bone	1	3	2	1	1	0	8
Nasal bone	7	16	13	14	4	2	56
Palatine bone	0	1	1	2	0	1	5
Zygomatic bone	5	13	23	11	7	2	61
Naso-orbito-ethmoidal	0	3	1	1	1	0	6
Orbital wall	0	3	0	0	1	0	4
Maxillary bone	4	4	12	8	3	3	34
Mandible	34	45	71	26	13	13	202
Total	51	88	123	63	30	21	376

**Figure 4.**
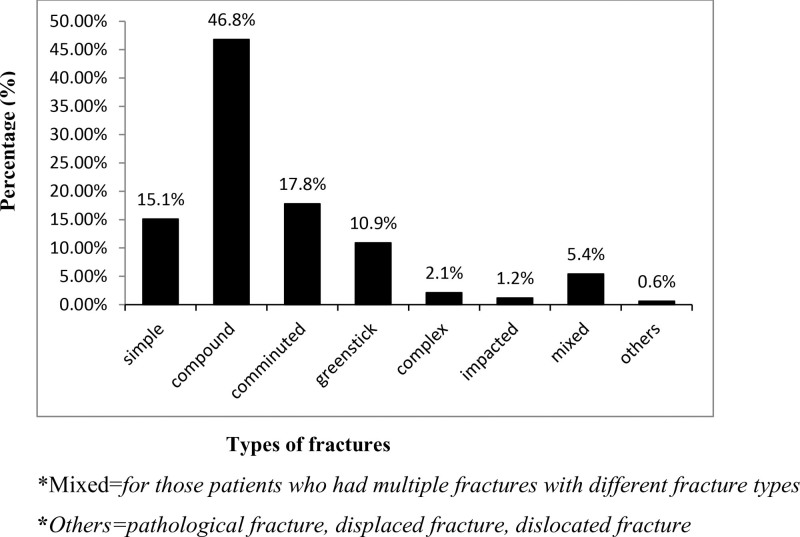
Types (nature) of maxillofacial bone fractures in patients who visited JMC dental clinic, 2018 to 2020.

Regarding the type (nature) of fracture, the majority of fractures were compound (open) type 155 (46.8%) followed by comminuted 59 (17.8%) (Fig. 4).

**Figure 5.**
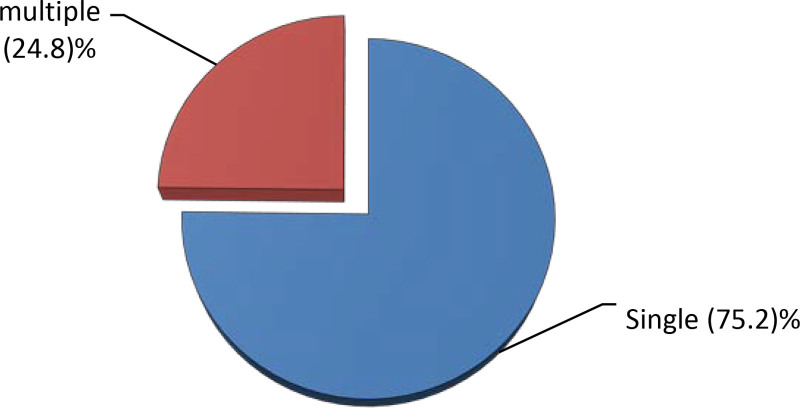
Type of fracture based on number of fractured maxillofacial bone in patients who visited JMC dental clinic, 2018 to 2020.

Two hundred forty-nine (75.2%) fracture cases had single fracture while the remaining 82 (24.8%) fracture cases had multiple fractures (Fig. 5).

Table 4 presents types (nature) of fractures by age group among maxillofacial fractures patients who visited JMC dental clinic from January 2018 to December 2020. Except for the 0 to 10 age groups, where greenstick fractures were the most common, compound (open) fractures were the most common type of fracture in all other age groups (Table 4).

**Table 4:** Types (nature) of fractures by age group among maxillofacial fractures patients who visited Jimma Medical Center dental clinic, 2018 to 2020.

Types of fractures	Age category						Total
	0 to 10	11 to 20	21 to 30	31 to 40	41 to 50	>50	
Simple	10	8	15	6	5	6	50
Compound	17	32	60	24	15	7	155
Comminuted	5	9	26	11	4	4	59
Greenstick	19	14	2	0	0	1	36
Complex	0	1	2	3	0	1	7
Impacted	0	1	2	1	0	0	4
Mixed	0	6	4	7	0	1	18
Others	0	0	2	0	0	0	2
Total	51	71	113	52	24	20	331

Table 5 presents the association between socio-demographic factors and number of fractured bones. In this study, number of fractured bones was not significantly associated with age (Pearson chi-square = 4.436, *P*-value = 0.489) or sex (Pearson chi-square = 0.002, *P*-value = 0.967). This means there was no statistically significant difference in the number of fractured bones between the 2 sex groups or age groups, regardless of whether they were single or multiple fractures (Table 5).

**Table 5:** Association between sociodemographic factors and number of fractured bones in maxillofacial fracture patients who visited Jimma Medical Center dental clinic, 2018 to 2020.

Variables	Number of fractured bone		Chi-square	*P*-value
	Single n (%)	Multiple n (%)		
Sex				
Male	210 (75.26)	69 (24.74)	0.002	.967
Female	39 (75)	13 (25)		
Age group				
0 to 10	40 (78.4)	11 (21.6)	4.436	.489
11 to 20	54 (76.1)	17 (23.9)		
21 to 30	81 (71.7)	32 (28.3)		
31 to 40	40 (76.9)	12 (23.1)		
41 to 50	16 (66.7)	8 (33.3)		
>50	18 (90)	2 (10)		

#### 4.2.1. Etiology of maxillofacial fractures

The leading cause of maxillofacial fractures in this study was road traffic accidents (RTA), accounting for 149 (45%) followed by assault 110 (33.2%) and falling down accident 39 (11.8%). Other causes of maxillofacial fractures, such as sports injuries, gunshot, animal strikes, and occupational injuries, were rare in this study, accounting for only 33 (10%) of all fractures.

Except for the nasal bone, which was mostly fractured by assault, RTA were the most common cause of fractures in all other facial bones (Table 6).

**Table 6:** Etiology and anatomical site of fractures in patients with maxillofacial bone fracture who visited Jimma Medical Center dental clinic, 2018 to 2020.

Anatomical sites	Frequency	Percentage (%)
Symphysis	34	10.5
Parasymphysis	88	27.2
Body	68	21
Angle	48	14.8
Ramus	5	1.57
Coronoid process	4	1.23
Condylar process	38	11.7
Dentoalveolar process	39	12
Total	324	100

Road traffic accident was the leading cause of injury across all age groups, with the exception of children aged 0 to 10, where accidental falls were the most common cause of maxillofacial bone fractures (Table 7).

**Table 7:** The distribution of fracture etiology across age groups in patients with maxillofacial fractures who visited the Jimma Medical Center dental clinic, 2018 to 2020.

Age group	Etiology of fractures				Frequency	Percentage (%)
	RTA	Fall	Assault	Others*		
0 to 10	6	26	6	13	51	15.4
11 to 20	41	2	24	4	71	21.5
21 to 30	60	2	47	4	113	34.1
31 to 40	23	2	21	6	52	15.7
41 to 50	13	2	6	3	24	7.3
>50	6	5	6	3	20	6
Total	149	39	110	33	331	100

* Others = other causes of fractures such as sports, work related, animal strike, gunshot etc.

In this study, the etiology of fractures was significantly associated with age (Pearson chi-square = 135.075, *P*-value = .000), sex (Pearson chi-square = 21.866, *P*-value = .000) and number of fractured bones (Pearson chi-square = 26.998, *P*-value = .000). These means there was a statistically significant difference in the causes of fractures across age groups, sex and number of fractures. Males were more likely than females to be involved in RTAs and assaults. With the exception of children aged 0 to 10, where accidental falls were the most common causes of injury, RTA was the leading cause of injury across all age groups. Multiple fractures were more common in RTA cases than in assaults and falls, where single fractures were more common (Table 8).

**Table 8:** Association of etiology of fractures with age, sex, and number of fractured bones in patients with maxillofacial fractures who visited Jimma Medical Center dental clinic, 2018 to 2020.

Variables	Etiology of fractures				Chi-square	*P*-value
	RTA n (%)	Fall n (%)	Assault n (%)	Others n (%)		
Sex						
Male	128 (45.9)	32 (11.5)	100 (35.8)	19 (6.8)	21.866*	0.000
Female	21 (40.4)	7 (13.5)	10 (19.2)	14 (26.9)		
Age groups						
0 to 10	6 (11.8)	26 (51)	6 (11.8)	13 (25.5)	135.075*	0.000
11 to 20	41 (57.7)	2 (2.8)	24 (33.8)	4 (5.6)		
21 to 30	60 (53.1)	2 (1.8)	47 (41.6)	4 (3.5)		
31 to 40	23 (44.2)	2 (3.8)	21 (40.4)	6 (11.5)		
41 to 50	13 (54.2)	2 (8.3)	6 (25)	3 (12.5)		
>50	6 (30)	5 (25)	6 (30)	3 (15)		
Number of fractured bone						
Single	93 (37.3)	31 (12.4)	93 (37.3)	32 (12.9)	26.998*	.000
Multiple	56 (68.3)	8 (9.8)	17 (20.7)	1 (1.2)		

* Others = other causes of fractures such as sports, work related, animal strike, gunshot, etc.

#### 4.2.2. Associated injuries

Of the total 331 patients studied, 31 (9.4%) of patients had associated injuries. Among this 16 (51.6%) of these had head injuries, 7 (22.6%) had cervical spine injuries, 3 (9.7%) had thoracic and extremity injuries, each respectively, and 2 (6.4%) were abdominopelvic injuries.

### 4.3. Posttreatment complications

Of the 331 maxillofacial fracture patients, only 23 (6.8%) cases developed complication after they had been treated. The most common complication was surgical site infection, which was developed in 12 (52.2%) cases. Malocclusion, non-union and other complications (ankylosis, chronic sinusitis, osteomyelitis etc), were developed only in 3 (13.0%), 2 (8.7%) and 6 (26.1%), patients respectively (Table 9).

**Table 9:** Type of posttreatment complications in patients with maxillofacial bone fractures who visited Jimma Medical Center dental clinic, 2018 to 2020.

Complications	Frequency	Percent (%)
Infection	12	52.2
Malocclusion	3	13.0
Non-union	2	8.7
Others	6	26.1
Total	23	100

## 5. Discussion

The etiological factors and pattern of maxillofacial fractures have been reported to vary from 1 geographical area to another depending on socioeconomic status, geographic location, and cultural characteristics^[5,10,21,22]^ This study attempted to determine the etiology and pattern of maxillofacial fractures in patients who visited the JMC dental clinic during the study period. It was conducted 3 years retrospectively on 331 maxillofacial fracture cases.

The sex distribution of maxillofacial fracture in this study is highly prevalent in males, which is consistent with the findings of other studies.^[11–13,23]^ The overall male-to female ratio in this study is 5.36:1. This ratio is comparable with a study conducted in, Kuwait^[14]^ and Italy^[15]^ 5.4:1, each respectively. This may be because men participate in the majority of outdoor activities in most countries, while most women, especially in rural areas, are confined to housework. It may also be due to the fact that more men drive vehicles and ride motorcycles, as well as the fact that drug and alcohol abuse is more common in men.^[13,24,25]^

On the contrary, in countries such as Austria^[13]^ and Canada,^[17]^ the male-to-female ratio of patients with maxillofacial fractures was low (2.1:1 and 1.6:1, respectively). This low ratio may attribute to the fact that females in those countries actively participate in social activities, making them more vulnerable to RTA and urban violence.

Our data showed that most of the patients were between the ages of 21 to 30 (34.1%). This finding is consistent with a study conducted in China^[26]^ and India,^[16]^ where the most affected groups were in between the ages of 21 to 30 (32.7% and 34.9%, respectively). The possible reason for this finding may be due to the fact that these age groups represent the most productive and reproductive age group and therefore economically and socially active, participating in higher levels of economic and high-risk activities such as dangerous driving, violence, and commercial use of automobiles. However, in comparison to a study conducted in France (45%),^[27]^ the current study’s prevalence for this age group was relatively low. The reason for the difference observation may be due to the difference in sample size and study period. In this study, the mandible was the most prevalent site for facial fractures in both genders (64.54%). This finding is in line with study conducted in France (65%),^[27]^ Saudi Arabia^[28]^ (56.6%) and Libya (58%).^[29]^ The finding of our study is relatively lower than that of study conducted in India (72%).^[30]^ This high prevalence in the Indian study may be attributed to its large sample size (n = 707) and study populations, which only included adults aged 18 to 65. On the other hand, the prevalence of mandibular fracture in our study is higher than the study conducted in Gonder, Ethiopia (32.9%).^[18]^ This discrepancy is may be due to the difference in the study populations.

However, contrary to our findings, the midface was identified as the most frequently fractured site in studies conducted in Malaysia,^[31]^ Germany^[32]^ and Portuguese.^[33]^ The fact that the mandible is more frequently involved in maxillofacial fracture is due to the fact that it is the most prominent and only moveable facial bone, and thus has a greater chance of being fractured than the well-articulated mid-facial bones. It may also be due to its mechanically weak components such as the angle, condylar neck, and both sides of the mentum, which can be fractured with minimal force of injury. It may also be because victims may put the mandible at a higher risk of impact during an accident while they are trying to avoid injury to the head.^[1,6]^

In our study, the most common mandibular fractured location was parasymphysis (27.2%), which is agree with of study conducted in India (37%)^[34]^ and (45.3%).^[35]^ On the contrary, the most common mandibular fractured location in studies conducted in Brazil^[36]^ and Germany^[37]^ was the condyle (29.98% and 42%), which is the fifth most common fractured site of the mandible in our study. Angle (35.6%, 32% and 22%, respectively) was the most commonly fractured site in studies conducted in the United States,^[38]^ Tasmania^[39]^ and Egypt,^[40]^ and it is the third most common fractured site of the mandible in our series. In studies conducted in Iran,^[41]^ Nigeria^[42]^ and Kenya,^[43]^ body was the most fractured site (41.77%, 29.6% and 26%, respectively), which is the second most common fractured site of the mandible in our study. These differences in mandibular fractured location may be attributed to mechanism of injury, direction of the force of injury and differences in sample size used in those studies.

RTA (45%) was found to be the most common cause of maxillofacial fractures in this study, which is consistent with the findings of studies conducted in China (42.2%),^[26]^ Turkey (43.95%),^[44]^ Nigeria (46.5%)^[45]^ and Egypt (40.9%).^[46]^ This is may be attributed to the driver’s recklessness, driving under the influence of alcohol or drugs and disregard for traffic laws, overspeeding, overloading, underage driving, and poor road and vehicle conditions. Alcoholism is strongly linked to facial injuries.^[47]^ In contrast, an assault was reported as the most common cause of maxillofacial fracture in studies conducted in Romania,^[48]^ Sweden,^[49]^ France^[27]^ and South Africa.^[50]^ These etiological differences may be attributed to differences in socioeconomic factors, national infrastructure development (especially roadways, traffic regulations, and legislation), and other behavioral practices such as alcohol consumption and other criminal activities.^[47,51]^

In contrast to our study, the study conducted in Gonder, Ethiopia^[18]^ was reported interpersonal violence as most common cause of maxillofacial fractures. The reason for the difference observation may be due to the tremendous increase in the number of vehicles in the country from time to time. The Gonder was conducted about 3 years (September 2013—August 2015) prior to our study.

In this study, concomitant head injury (51.61%) is the most common associated injury with maxillofacial fractures, which was supported by studies conducted in India (56.1%)^[16]^ and Libya (33.3%).^[29]^ This may be because of its anatomical close proximity to maxillofacial areas. In contrast to a study conducted in Gonder^[18]^ that found a high number (29.1%) of extremity injuries in association with maxillofacial fractures, the current study found a low number (9.68%). This may be because interpersonal violence was the leading cause of fracture in the Gonder study; as a result, victims may have used their arms to defend themselves against assaults.

In our study, the postoperative complication rate was found to be 6.8% which is similar to the findings of study in China^[26]^ (7.2%) and Turkey^[52]^ (6%), but lower than that found by study conducted in Uganda^[53]^ (29.54%) and Gonder, Ethiopia^[18]^ (15.2%). These disparities in complication rates between studies may be attributed to the severity of the fractures and the procedures used to treat the patients. Closed reduction was used in the majority of those with high complication rates, which could be due to a lack of qualified maxillofacial surgeons and/or a scarcity of materials used to perform open reduction procedures.

## 6. Limitation of the study

The retrospective nature of this study has inherent limitations due to incomplete records, gaps in information, and information obtained based on assessment and documentation by various medical professionals. As a result, it was difficult to broaden the scope of this study. There were other variables that were not studied that might have an influence for the causes of fractures. This study was done in single center; hence the findings may fail to reflect the true picture of maxillofacial fractures in the whole country at large.

## 7. Conclusion

According to this study, most of the patients were young adult males in their third decade of life. The main etiological factor was RTA, followed by assault and accidental fall. Mandible was the most frequently fractured facial bone, with parasymphysis being the most common site. Head and cervical spine injuries were the most common associated injuries. Surgical site infection was the most common posttreatment complication. Age, sex and number of fractured bones have significant association with etiology of fracture. The findings of this study can be used to guide public health activities, healthcare professional training, and resource allocation in Ethiopia in order to enhance maxillofacial fracture prevention, management, and outcomes.

## Acknowledgments

Firstly, we would like to express our deepest gratitude to the Jimma University for ethical approval. Our gratitude and appreciation go to data collectors, supervisors, Jimma Medical Center for their cooperation during data and study participants without their cooperation this study would have not been possible.

## Author contributions

**Conceptualization:** Gemechu Tola Wayiso.

**Data curation:** Gemechu Tola Wayiso, Midekso sento Erba, Diliab Desta.

**Formal analysis:** Gemechu Tola Wayiso.

**Funding acquisition:** Gemechu Tola Wayiso.

**Investigation:** Gemechu Tola Wayiso, Fikadu Seyoum Tola.

**Methodology:** Gemechu Tola Wayiso, Midekso sento Erba.

**Visualization:** Fikadu Seyoum Tola.

**Writing – original draft:** Gemechu Tola Wayiso.

**Writing – review & editing:** Gemechu Tola Wayiso.

## References

[1] Moore KL Dalley AF II Agur AMR. Clinically oriented anatomy. 7th ed. Lippincott Williams & Wilkins; 2014.

[2] Angelopoulos C. Anatomy of the maxillofacial region in the three planes of section. Dent Clin North Am. 2014;58:497–521. 24993921 10.1016/j.cden.2014.03.001

[3] Pillay L Mabongo M Buch B. Prevalence and aetiological factors of maxillofacial trauma in a rural district hospital in the Eastern Cape. South Afr Dent J. 2018;73:348–53.

[4] Gómez Roselló E Quiles Granado AM Artajona Garcia M. Facial fractures: classification and highlights for a useful report. Insights Imaging. 2020;11:49. 32193796 10.1186/s13244-020-00847-w PMC7082488

[5] Naveen Shankar A Naveen Shankar V Hegde N Sharma Prasad R. The pattern of the maxillofacial fractures – a multicentre retrospective study. J Craniomaxillofac Surg. 2012;40:675–9. 22212823 10.1016/j.jcms.2011.11.004

[6] DMD RJF. Oral and Maxillofacial Surgery. 3rd ed. Saunders; 2017:2696.

[7] Penmetsa GS Kavyamala Dubba ZM. Prevalence and pattern of maxillofacial trauma in north chennai: a retrospective study. J Indian Assoc Public Health Dent. 2018;16:230–3.

[8] Rajanikanth K Borle RM Bhola N. The pattern of maxillofacial fractures in central India a unicentric retrospective study. IOSR J Dent Med Sci. 2014;13:28–31.

[9] Al-Bokhamseen M Salma R Al-Bodbaij M. Patterns of maxillofacial fractures in Hofuf, Saudi Arabia: a 10-year retrospective case series. Saudi Dent J. 2018;31:129–36. 30705576 10.1016/j.sdentj.2018.10.001 PMC6349956

[10] Mesgarzadeh AH Shahamfar M Azar SF Shahamfar J. Analysis of the pattern of maxillofacial fractures in north western of Iran: a retrospective study. J Emerg Trauma Shock. 2011;4:48–52. 21633568 10.4103/0974-2700.76837 PMC3097580

[11] Motamedi MHK Dadgar E Ebrahimi A Shirani G Haghighat A Jamalpour MR. Pattern of maxillofacial fractures: a 5-year analysis of 8,818 patients. J Trauma Acute Care Surg. 2014;77:630–4. 25250606 10.1097/TA.0000000000000369

[12] Almasri M. Severity and causality of maxillofacial trauma in the Southern region of Saudi Arabia. Saudi Dent J. 2013;25:107–10. 24179319 10.1016/j.sdentj.2013.04.001 PMC3809494

[13] Gassner R Tuli T Hächl O Rudisch A Ulmer H. Cranio-maxillofacial trauma: a 10 year review of 9543 cases with 21 067 injuries. Craniomaxillofac Surg. 2003;31:51–61. 10.1016/s1010-5182(02)00168-3 12553928

[14] Schütz P Safar S Al-Yassin SM Belal MS Korinek P. Maxillofacial fractures in Kuwait between 1992-1997. Asian J Oral Maxillofac Surg. 2001;13:195–201.

[15] Arangio P Vellone V Torre U Calafati V Capriotti M Cascone P. Maxillofacial fractures in the province of Latina, Lazio, Italy: review of 400 injuries and 83 cases. J Craniomaxillofac Surg. 2013;42:583–7. 24035287 10.1016/j.jcms.2013.07.030

[16] Gandhi S Ranganathan LK Solanki M Mathew GC Singh I Bither S. Pattern of maxillofacial fractures at a tertiary hospital in northern India: a 4-year retrospective study of 718 patients. Dent Traumatol. 2011;27:257–62. 21635691 10.1111/j.1600-9657.2011.00996.x

[17] Al-Dajani M Quiñonez C Macpherson AK Clokie C Azarpazhooh A. Epidemiology of maxillofacial injuries in Ontario, Canada. J Oral Maxillofac Surg. 2015;73:693.e1–9. 10.1016/j.joms.2014.12.001 25661507

[18] Teshome A Andualem G Tsegie R Seifu S. Two years retrospective study of maxillofacial trauma at a tertiary center in North West Ethiopia. BMC Res Notes. 2017;10:1–6. 28789668 10.1186/s13104-017-2670-1 PMC5549360

[19] Tugaineyo EI Odhiambo WA Akama MK Guthua SW Dimba EAO. Aetiology, pattern and management of oral and maxillofacial injuries at mulago national referral hospital. East Afr Med J. 2012;89:351–8. 26852446

[20] Boffano P Roccia F Zavattero E. European Maxillofacial Trauma (EURMAT) project: a multicentre and prospective study. J Craniomaxillofac Surg. 2015;43:62–70. 25457465 10.1016/j.jcms.2014.10.011

[21] Chrcanovic BR Abreu MHNG Freire-Maia B Souza LN. 1,454 mandibular fractures: a 3-year study in a hospital in Belo Horizonte, Brazil. J Craniomaxillofac Surg. 2012;40:116–23. 21458284 10.1016/j.jcms.2011.03.012

[22] Zaleckas L Pečiuliene V Gendviliene I Puriene A Rimkuviene J. Prevalence and etiology of midfacial fractures: a study of 799 cases. Medicina (Kaunas). 2015;51:222–7. 26424186 10.1016/j.medici.2015.06.005

[23] Kieser J Stephenson S Liston PN. Evidence based medicine: trauma Serious facial fractures in New Zealand from 1979 to 1998. Int J Oral Maxillofac Surg. 2002;31:206–9. 12102421 10.1054/ijom.2002.0208

[24] Ravindran V Ravindran Nair KS. Metaanalysis of maxillofacial trauma in the Northern Districts of Kerala: one year prospective study. J Maxillofac Oral Surg. 2011;10:321–7. 23204748 10.1007/s12663-011-0264-3 PMC3267926

[25] Shenoi SR Budhraja N Badjate S. An assessment of maxillofacial fractures: a two-year retrospective study. J Emerg Trauma Shock. 2012;5:205. 22787359 10.4103/0974-2700.96506 PMC3391853

[26] Mijiti A Ling W Tuerdi M. Epidemiological analysis of maxillofacial fractures treated at a university hospital, Xinjiang, China: a 5-year retrospective study. J Craniomaxillofac Surg. 2013;42:227–33. 23791439 10.1016/j.jcms.2013.05.005

[27] Pham-Dang N Barthélémy I Orliaguet T Artola A Mondié JM Dallel R. Etiology, distribution, treatment modalities and complications of maxillofacial fractures. Med Oral Patol Oral Cir Bucal. 2014;19:261–9. 10.4317/medoral.19077 PMC4048115 24316696

[28] Abdullah WA Al-Mutairi K Al-Ali Y Al-Soghier A Al-Shnwani A. Patterns and etiology of maxillofacial fractures in Riyadh City, Saudi Arabia. Saudi Dent J. 2013;25:33–8. 23960553 10.1016/j.sdentj.2012.10.004 PMC3723071

[29] Elarabi MS Bataineh AB. Changing pattern and etiology of maxillofacial fractures during the civil uprising in Western Libya. Med Oral Patol Oral Cir Bucal. 2018;23:e248–55. 29476683 10.4317/medoral.22268 PMC5911362

[30] Anitha R Devakumari S Dinesh DS Canmany E Devameena S. Prevalence and patterns of maxillofacial trauma in South India—a retrospective study for seven years. J Contemp Med Dent. 2020;8:54–7.

[31] Abosadegh MM Saddki N Al-Tayar B Rahman SA. Epidemiology of maxillofacial fractures at a teaching hospital in Malaysia: a Retrospective Study. Biomed Res Int. 2019;2019:1–10. 10.1155/2019/9024763 PMC6393910 30895196

[32] Schneider D Kämmerer PW Schön G Dinu C Radloff S Bschorer R. Etiology and injury patterns of maxillofacial fractures from the years 2010 to 2013 in Mecklenburg-Western Pomerania, Germany: a retrospective study of 409 patients. J Craniomaxillofac Surg. 2015;43:1948–51. 26427620 10.1016/j.jcms.2015.06.028

[33] Alves LS Aragão I Sousa MJC Gomes E. Pattern of maxillofacial fractures in severe multiple trauma patients: a 7-year prospective study. Braz Dent J. 2014;25:1–4. 10.1590/0103-6440201302395 25590206

[34] Ragupathy K Pasupathy S. Incidence, aetiology and pattern of mandibular fractures in Pondicherry. J Evol Med Dent Sci. 2015;4:16946–50.

[35] Sirimaharaj W Pyungtanasup K. The epidemiology of mandibular fractures treated at Chiang Mai University Hospital: a review of 198 cases. J Med Assoc Thailand. 2008;91:868–74. 18697387

[36] Chrcanovic BR. Factors influencing the incidence of maxillofacial fractures. Oral Maxillofac Surg. 2012;16:3–17. 21656125 10.1007/s10006-011-0280-y

[37] Bormann KH Wild S Gellrich NC. Five-year retrospective study of mandibular fractures in Freiburg, Germany: incidence, etiology, treatment, and complications. J Oral Maxillofac Surg. 2009;67:1251–5. 19446212 10.1016/j.joms.2008.09.022

[38] Boole JR Holtel M Amoroso P Yore M. 5196 Mandible fractures among 4381 active duty army soldiers, 1980 to 1998. Laryngoscope. 2001;111:1691–6. 11801927 10.1097/00005537-200110000-00004

[39] Dongas P Hall GM. Mandibular fracture patterns in Tasmania, Australia. Aust Dent J. 2002;47:131–7. 12139266 10.1111/j.1834-7819.2002.tb00316.x

[40] Sakr K Farag IA Zeitoun IM. Review of 509 mandibular fractures treated at the University Hospital, Alexandria, Egypt. Br J Oral Maxillofac Surg. 2006;44:107–11. 15896887 10.1016/j.bjoms.2005.03.014

[41] Khorasani M Khorasani B. The epidemiology of mandibular fractures in Qazvin Province, Iran: a retrospective study (1995-2005). Res J Biol Sci. 2009;4:738–42.

[42] Adeyemo WL Iwegbu IO Bello SA. Management of mandibular fractures in a developing country: a review of 314 cases from two urban centers in Nigeria. World J Surg. 2008;32:2631–5. 18841410 10.1007/s00268-008-9773-8

[43] Owino R Macigo F Onyango F. Pattern and aetiology of mandibular fractures at Kenyatta National Hospital. Afr J Oral Health Sci. 2003;4:178–80.

[44] Ortakoǧlu K Günaydin Y Aydintuǧ YS Bayar GR. An analysis of maxillofacial fractures: A 5-year survey of 157 patients. Mil Med. 2004;169:723–7. 15495729 10.7205/milmed.169.9.723

[45] Udeabor SE Akinbami BO Yarhere KS Obiechina AE. Maxillofacial fracture etiology pattern of presentation and treatment. J Dent Surg. 2014;2014:1–6.

[46] Mabrouk A Helal H Mohamed A Mahmoud N. Incidence, etiology, and patterns of maxillofacial fractures in Ain-Shams University, Cairo, Egypt: a 4-year retrospective study. Craniomaxillofac Trauma Reconstr. 2014;7:224–32. 25136412 10.1055/s-0034-1374061 PMC4130756

[47] Jessica B Angus C Lara F Steve Evans BW Thomson M. Maxillofacial fractures at Waikato Hospital, New Zealand: 1989 to 2000. N Z Med J. 2005;118:1–9. 15980903

[48] Tent PA Popa D Juncar R Haranguş A Lung T Juncar M. Traumatic causes of mandibular fractures – a 3-year prospective clinical study. Hum Vet Med. 2017;9:48–52.

[49] Sand L Gavelin P Hirsch J Ramadhan A. A retrospective study of patients with mandibular fractures treated at a Swedish University Hospital 1999-2008. Ann Maxillofac Surg. 2014;4:178. 25593868 10.4103/2231-0746.147119 PMC4293839

[50] Roode GJ van Wyk PJ Botha SJ. Mandibular fractures: an epidemiological survey at the oral and dental hospital, Pretoria. SADJ. 2007;62:270, 272–4. 17927035

[51] Cheema SA Amin F. Incidence and causes of maxillofacial skeletal injuries at the Mayo Hospital in Lahore, Pakistan. Br J Oral Maxillofac Surg. 2006;44:232–4. 16054279 10.1016/j.bjoms.2005.05.017

[52] Özkaya O Turgut G Kayali MU Urlu KU Bafi L. A retrospective study on the epidemiology and treatment of maxillofacial fractures. Turkish J Trauma Emerg Surg. 2009;15:262–6. 19562549

[53] Kamulegeya A Lakor F Kabenge K. Clinical science oral maxillofacial fractures seen at a Ugandan Tertiary Hospital: a six-month prospective study. Clinics (Sao Paulo). 2009;64:843–8. 19759877 10.1590/S1807-59322009000900004 PMC2745137

